# Quality of postnatal care for mothers and neonates in Mexico: Insights from the maternal eCohort study

**DOI:** 10.1371/journal.pone.0323039

**Published:** 2025-08-12

**Authors:** Martín Paredes-Cruz, Diana Perez-Moran, Svetlana V. Doubova, Catherine Arsenault, Claudio Quinzaños-Fresnedo

**Affiliations:** 1 Epidemiology and Health Services Research Unit CMN Siglo XXI, Mexican Institute of Social Security, Mexico City, Mexico; 2 Department of Global Health. Milken Institute School of Public Health, The George Washington University, Washington District of Columbia, United States of America; 3 Dirección de Prestaciones Médicas, Instituto Mexicano del Seguro Social, Ciudad de México, México; National Research Centre, EGYPT

## Abstract

**Objective:**

The study aimed to evaluate healthcare use during the postnatal period for mothers and their babies, the content of care received, mothers’ perceived quality of care, and the factors influencing these perceptions.

**Methods:**

The study analyzed data from a postnatal survey conducted within the maternal eCohort at the Mexican Institute of Social Security (IMSS). The research involved 973 women aged 18–49 who were recruited following their first antenatal care visit with a family physician at 48 family medicine clinics across eight Mexican states. We described postnatal care (PNC) use, content of care and perceived quality and used Poisson multivariable regression analysis to investigate the factors influencing women’s perceptions of higher quality of care during PNC.

**Results:**

29.4% of women and 12.0% of infants had no postnatal care visits within the six weeks following hospital discharge. Among women who received PNC, 72.3% accessed services exclusively through IMSS, 17.7% utilized a combination of IMSS and other providers, and 10% relied solely on private providers. Infants received 82.4% of recommended content of care, compared to 66.7% for mothers. The median perceived quality of care among women was 25 points on a scale of 8–40. Key areas for improvement include enhancing awareness of the importance of postnatal consultations among health personnel and women, reducing waiting times, and improving the content and length of consultations.

Factors associated with better perceived quality included being over 35, receiving better content of care for infants, and being treated by private providers, while lower education levels, prior pregnancies, and poor health were associated with lower perceived quality.

**Conclusion:**

Improvements are needed to ensure all women and infants receive comprehensive postnatal care and to enhance patient-perceived quality.

## Introduction

Postnatal care (PNC) is an important part of the health care continuum for mothers, newborns, and infants. It should prioritize the prevention, timely diagnosis, and treatment of childbirth-related complications to reduce morbidity and mortality while promoting the well-being of mother-child pairs through evidence-based, person-centered care [1]. This is why the World Health Organization (WHO) and the Pan American Health Organization (PAHO) recommend three postnatal check-ups by health professionals within the first six weeks after childbirth [1].

Despite the essential role of PNC worldwide, median coverage rates reveal concerning gaps: only 71% of mothers and 64% of infants receive PNC [2]. Across 23 low- and middle-income countries, a study estimated that only 41.4% of women and 42.3% of newborns received high-quality postnatal care [3]. However, existing data on the quality of postnatal care (PNC) are largely drawn from studies in Africa, Southeast Asia, the Eastern Mediterranean, and Europe, with limited or no information available from North American and Latin American countries [3].

The Mexican Institute of Social Security (IMSS) is recognized as the largest healthcare provider in Mexico and Latin America, serving over 74 million people, primarily workers from the formal labor sector and their families. At IMSS, PNC is provided by family physicians and nurses at primary care clinics. This care follows official Mexican standards [4], which recommend at least two postnatal check-ups – one at 15 days and another at 6 weeks postpartum – and aligns with WHO guidelines on PNC processes. In 2024, over 300,000 mothers delivered their babies in IMSS hospitals. Despite a high number of births, IMSS lacks official data on PNC utilization and quality. Limited studies on this topic have revealed that only around half of mothers seek PNC. This is primarily due to a low perception of the need for PNC [5,6]. Yet, there is no information on the quality of postnatal care at IMSS.

Therefore, the objective of the present study was to assess mother and infant PNC utilization, PNC content and perceived quality, and to examine the factors associated with women’s perceptions of PNC quality.

## Methods

The study analyzed data from a postnatal survey conducted within the maternal eCohort at the IMSS [7]. The cohort enrolled 1,390 pregnant women aged 18–49 years after receiving their first antenatal care consultation with a family physician at 48 IMSS family medicine clinics across eight states in four regions of Mexico. The participating states were Aguascalientes and Jalisco in the West; Coahuila and Nuevo León in the North; Veracruz and Yucatán in the Southeast; and the State of Mexico and Mexico City in the Central region. These states were selected by convenience, focusing on those with the highest number of first antenatal care visits. Participant recruitment took place from August to December 2023, followed by subsequent follow-up until August 2024. Detailed information on the cohort selection criteria, sample size, and sampling strategy have been previously published [7].

The survey questionnaire was designed by the Quality Evidence for Health System Transformation (QuEST) network to assess PNC quality from women’s perspective. Prior to data collection, the study questionnaire was adapted to the Mexican context and validated by IMSS experts [7]. The postnatal interviews were conducted between 6 and 8 weeks postpartum, based on women’s time availability. In the present study, we defined the postnatal period as the 6 weeks following hospital discharge after childbirth.

### Study variables

To describe the study population, we collected information on women’s sociodemographic characteristics including age, education level, marital status, employment, primiparity, and region of residence (West (Aguascalientes and Jalisco), North (Coahuila and Nuevo León), Southeast (Veracruz and Yucatán), and Center (State of Mexico and Mexico City) regions).

Covariates on infants’ and women’s health status included: the mother’s perception of the child’s health (excellent, very good, good, regular and poor); infant health problems after hospital discharge and up until the time of the survey (i.e., feeding, bowel movement and sleep problems, as well as skin alterations), danger signs (i.e., presence of disease with a cough, trouble breathing, fever (body temperature > 37.5 ºC), experience of diarrhea with blood in the stools, hypothermia, seizures, jaundice), and current child feeding (exclusive breastfeeding, formula, both). Women’s health status was measured using self-rated health on a five-point Likert scale (excellent, very good, good, regular and poor). Postpartum depression was assessed using the Patient Health Questionnaire (PHQ-9) which was validated in Mexico [8]. This scale has nine questions and four response options ranging from 0 (none of the days) to 3 (almost every day), with a grand total ranging from 0 to 27. The severity of the symptoms is organized into six categories of depression risk: 0−4 (minimal), 5−9 (mild), 10−14 (moderate), 15−19 (moderate to severe) and 20−27 (severe). For the purpose of this study and considering the low prevalence of women who scored 10 or above on the PHQ-9, we defined the risk of depression as a score of 5 or more.

#### PNC utilization.

PNC utilization was assessed using a categorical variable for the number and type of medical consultations by the mother-infant pairs (none, mothers only, infants only, or both mothers and babies). We also assessed the total number of consultations for mothers and infants and the number of postnatal check-ups for mothers and babies or other types of consultations with health professionals during this period (i.e., consultations related to health problems, neonatal screening, vaccinations, or other concerns). Women also reported the type of healthcare providers used (such as IMSS or other providers like private provider or the Ministry of Health, or use of both IMSS and other providers) and the potential reasons for not receiving care in the postnatal period.

#### Content of PNC for infants.

We surveyed women on clinical activities performed by health providers for the baby (measuring weight, height, head, chest, abdomen and body temperature, listening to the chest with a stethoscope, examination of eyes, hearing, reflexes, genitals and hip bones), advices on baby care including vaccines that the baby should receive, danger signs that require medical care, breastfeeding and its frequency, safe sleeping positions, cases that require follow-up with a pediatrician or a neonatologist and how to play and interact with the baby. We also generated a variable that describes the percentage of activities provided to the baby regarding required PNC clinical activities, including all previously defined activities, with a minimum score of 0% and maximum of 100%. In addition, we collected information on the health provider’s recommendations regarding any infant danger signs and current baby’s vaccination status for the BCG vaccine, pentavalent vaccine, pneumococcal vaccine and rotavirus vaccine.

#### Content of PNC for women.

PNC content for mothers included blood pressure and body temperature measurements, C-section scar examination (for women who had a c-section), and maternal counseling or advice (on danger signs during the postnatal period, mood, breast care, physical activity, on reinitiating sexual activity after childbirth, on family planning options and on emotional well-being). Furthermore, we created a score for the percentage of activities provided to each woman out of the total recommended.

#### Dependent variable.

**Perceived quality:** Women’s perception about quality of care during medical consultations in the postnatal period was measured by asking them to rate eight components of their care on a scale of 1–5 (poor, fair, good, very good, and excellent): (i) the level of respect shown by the physician; (ii) courtesy and helpfulness of non-medical staff; (iii) the extent to which the physician involved women in decisions about their care or care of their child; (iv) availability of medical equipment or access to laboratory tests; (v) clarity of the physician’s explanations; (vi) knowledge and skills of the physician; (vii) the amount of time the physician spent with the woman; and (viii) waiting time [7]. We calculated a summary score of perceived PNC quality as the sum of the response scores for each of the eight indicators. The score ranged from a minimum of 8 to a max of 40 points [7].

#### Sample size.

Detailed information regarding the cohort’s sample size, and sampling strategy have been published previously [7]. However, out of 1,390 women who agreed to participate in the maternal eCohort in Mexico and completed the baseline questionnaire, only 973 (70%) answered the postnatal care survey. Among the rest, 3.7% (n = 51) experienced a miscarriage, 0.6% had a stillbirth (n = 5) or neonatal death (n = 3), and 25.7% (n = 358) dropped out. The sample of 973 participants is sufficient to address the present study objectives, as it allows meeting the assumption of ensuring at least ten participants per covariate included in the multivariable regression analysis [9].

### Statistical analysis

We used descriptive statistics to describe women and infants’ health and demographic characteristics, PNC utilization, content of care, and perceived quality. We used percentages for categorical variables, and median with range (minimum and maximum values) for non-normally distributed numerical variables (verified by the Shapiro-Wilk test).

Second, to explore the factors associated with the perceived quality of PNC, we performed a multivariable Poisson regression analysis. We used Poisson regression because the dependent variable (perceived quality of care score) is a count variable ranging from 8 (indicating poor quality) to 40 points (indicating excellent quality), which aligns with the assumption of the Poisson distribution. Linear regression was not appropriate as the variable is not continuous. Ordinal logistic regression was also unsuitable, as it requires a categorical outcome with an inherent order or ranking. Our modeling strategy was based on the VanderWeele and Shpitser criteria for the selection of confounders [10]. These authors recommend including all conceptual and clinically relevant covariates to ensure that the final model adjusts for even mild confounders. In addition, the standard errors of the regression model were adjusted for clustering based on the women’s region of residence. The multivariable analysis on perceived PNC quality focused solely on women who had at least one consultation for themselves or their baby during the postnatal period (n = 901); however, because 16 participants (1.8%) had missing data for one or more study variables, they were automatically excluded from the analysis resulting in an analytic sample of 885 participants. Moreover, for the analysis of infant outcomes, we analyzed the information of one infant per woman because of the limited number of twins in the cohort (n = 12), and since the outcomes for the twins were similar, we only provided details about the firstborn twin.

A p-value of ≤0.05 was considered statistically significant. We analyzed the data using Stata 14 statistical software (Stata Corp LP; College Station, TX). The study is reported according to the STROBE (Strengthening the Reporting of Observational Studies in Epidemiology) guidelines.

### Ethics

The study was approved by the IMSS National Research and Ethics Committees (R-2022-785-064). Before participating in the study, all women provided written informed consent form.

## Results

### General characteristics and health status of mothers and infants

The majority of survey participants (86.1%) were 34 or younger, were married (84.7%), and had high school education (64.6%). More than half (60.4%) had remunerated jobs, and 36.5% were primiparous. Most participants were from the north (30.2%), followed by the central (25.9%), west (22.8%), and south (21.1%) regions (Table 1).

**Table 1:** General characteristics and health of mothers and infants.

General characteristics of mothers	n = 973 n (%)
**Age**	
≤34 years	838 (86.1)
≥ 35 years	135 (13.9)
**Schooling**	
Primary school or less	45 (4.6)
Secondary	299 (30.7)
High school or higher	628 (64.6)
Without information	1 (0.1)
**Marital status**	
Single/Divorced/Separated/Widowed	142 (14.6)
Married/Cohabiting	824 (84.7)
Without information	7 (0.7)
**Paid job**	588 (60.4)
**Primigravida**	355 (36.5)
**Region**	
North	294 (30.2)
Center	252 (25.9)
West	222 (22.8)
South	205 (21.1)
**Infant’s health**	**n = 973**
Mother’s perception of infant’s health as excellent, very good or good	955 (98.2)
Bowel movement problems	81 (8.3)
Sleep problems	80 (8.2)
Feeding problems	44 (4.5)
Respiratory problems	36 (3.7)
Skin alterations	28 (2.9)
**One or more infant’s danger signs**	135 (13.9)
A disease with a cough	65 (6.7)
Fever (temperature > 37.5 ºC)	41 (4.2)
Jaundice	33 (3.4)
Trouble breathing	21 (2.2)
Others (diarrhea with blood in the stools, hypothermia, seizures)	9 (0.9)
**Infant feeding**	
Exclusive breastfeeding	564 (58.0)
Both	327 (33.6)
Formula	82 (8.4)
**Women’s health**	**n = 973**
Women’s self-rated health as excellent, very good or good	921 (94.7)
Risk of postpartum depression	29 (3.0)

In the postnatal period, 98.2% reported their babies were in excellent or good health, with fewer than 10% facing some health issues such as bowel movement problems (8.3%), sleep difficulties (8.2%), feeding challenges (4.5%), respiratory issues (3.7%), or skin disorders (2.9%). In addition, 13.9% of babies exhibited danger signs, primarily cough (6.7%) and fever (4.2%). Regarding feeding practices, 58% of infants were exclusively breastfed, 33.6% received a combination of breastmilk and formula, and 8.4% used formula alone. Most women (94.7%) rated their health as excellent, very good, or good. Only 3% presented a risk of postpartum depression (Table 1).

### Healthcare use during the postnatal period

Following hospital discharge after childbirth, 7.4% of participants did not have any medical consultations. Of those who received care during the postnatal period, 4.6% of consultations were solely for women, 22% exclusively for infants, and 66% attended consultations for both mothers and their babies.

83.2% of infants attended at least one PNC visit, compared to 67.4% of mothers. One third (34.6%) had consultations for other needs, mainly for neonatal screening (46.9%), vaccinations (45.7%), or illnesses (19.3% for children and 11.0% for mothers) (Table 2). Most received postnatal care at IMSS (72.3%), while 17.7% used both IMSS and private services, and 10% relied solely on private healthcare providers. The primary reason for not seeking PNC was the belief that neither they nor their baby needed a consultation (40.3%), followed by appointment unavailability (19.4%) and long waiting times (12.5%) (Table 2).

**Table 2:** Healthcare use during the first 6 weeks after childbirth and discharge from the hospital.

	n = 973 n (%)
Use of medical consultations by mother-infant pair during the postnatal period	
None	72 (7.4)
For women only	45 (4.6)
For infants only	214 (22.0)
For both mothers and babies	642 (66.0)
Number of medical consultations for mothers	**n = 973**
0	286 (29.4)
1	525 (54.0)
2	111 (11.4)
3	51 (5.2)
Number of medical consultations for babies	**n = 973**
0	117 (12.0)
1	440 (45.2)
2	253 (26.0)
3	163 (16.8)
Postnatal check-up for babies	810 (83.2)
Postnatal check-up for women	656 (67.4)
Other types of consultations with a health professional in the postnatal period	337 (34.6)
Reasons for other consultations with a health professional in the postnatal period	**n = 337**
Neonatal screening	158 (46.9)
Vaccination	154 (45.7)
Baby’s health problem	65 (19.3)
Woman’s health problem	37 (11.0)
Other	58 (17.2)
Healthcare provider in the postnatal period	**n = 901**
IMSS	651 (72.3)
IMSS and health providers outside the IMSS	160 (17.7)
Health providers outside the IMSS (e.g., private, Ministry of Health)	90 (10.0)
Reason for not receiving health care in the postnatal period	**n = 72**
Baby and I don’t need it	29 (40.3)
Tried, but there were no appointments available, or the appointments were too far away	14 (19.4)
Long waiting time to obtain an appointment to see the doctor	9 (12.5)

### Content of PNC

During infant postnatal care visits, over 94% of women reported their babies’ being weighed, and their height and temperature measured. Additionally, 92.2% of infants had their chests examined with a stethoscope, while 81.3% had their eyes checked and 81.0% had their tendon reflexes assessed. At least 71.6% of babies had their genitals, hearing, and hip bones examined. Furthermore, most mothers received guidance on vaccinations (91.8%), danger signs for medical attention (85.8%), breastfeeding (81.9%), feeding frequency (74.4%), and safe sleeping positions (68.1%). Only 54.8% were advised on follow-up visits with a pediatrician, and 51.5% received advice on interacting with their babies. The median percentage of clinical activities provided to babies was 82.4%, ranging from 0 to 100%. Regarding the current baby’s vaccination status, 82.2% received the BCG vaccine, 55.9% the pentavalent vaccine, 50.3% the pneumococcal vaccine and only 48.2% the rotavirus vaccine (Table 3).

**Table 3:** Content of infant and women postnatal care in the first 6 weeks after childbirth.

Content of care	n = 856 n (%)
**Infant postnatal care**	
**Clinical activities performed for babies:**	
The weight was measured	832 (97.2)
The height, head, chest and abdomen were measured	814 (95.1)
The temperature was taken	807 (94.3)
The chest was listened to with a stethoscope	789 (92.2)
The eyes were examined	696 (81.3)
Reflexes were checked	693 (81.0)
Genitals were examined	613 (71.6)
The hearing was checked	612 (71.5)
Hip bones were examined	575 (67.2)
**Counseling on infant care**^¥^:	**n = 901**
Vaccines that the baby should receive	827 (91.8)
Danger signs in the baby	773 (85.8)
Breastfeeding	738 (81.9)
Feeding frequency	670 (74.4)
Safe sleeping positions	614 (68.1)
Cases that require follow-up with a pediatrician or a neonatologist	494 (54.8)
How to play and interact with the baby	464 (51.5)
**Percentage of clinical activities performed for infants**	**n = 901**
Average (SD)	75.5 (22.1)
Median (min-max)	82.4 (0-100)
**Health provider’s recommendations regarding infant danger signs** ^¥¥^	**n = 135**
Provided a prescription for the medication(s)	61(45.2)
Instructed to monitor the baby and return if it got worse	42 (31.1)
Told that it was normal	39 (28.9)
Recommended to go to the hospital to see a specialist	7 (5.2)
Recommended to get a lab test or imaging studies for the baby	9 (6.7)
Provided recommendation about feeding	11 (8.2)
Not discussed it with a doctor	6 (4.4)
**Infants’ vaccination status**	**n = 973**
BCG vaccine	800 (82.2)
Pentavalent vaccine	544 (55.9)
Pneumococcal vaccine	489 (50.3)
Rotavirus vaccine	469 (48.2)
**Women postnatal care** ^¥¥¥^	
**Clinical activities provided for women**	**n = 678**
Blood pressure measurement	630 (92.9)
Temperature measurement	591 (87.2)
C-section scar examination^¥¥¥¥^	**n = 557,** 338 (60.7)
**Counseling on women’s postnatal care:**	**n = 678**
Danger signs or symptoms during postnatal period	552 (81.4)
Family planning options after childbirth	503 (74.2)
Breast care	466 (68.7)
Importance of exercise or physical activity after childbirth	322 (47.5)
Reinitiating sexual activity after childbirth	316 (46.6)
Emotional well-being	293 (43.2)
Percentage of clinical activities provided for women	
Average (SD)	68.5 (28.0)
Median (min-max)	66.7 (0-100)

¥ All women, with any consultation during the postnatal period,

¥¥ Only babies with danger signs.

¥¥¥ Nine (1.3%) out of 687 women who received consultation during the postnatal period reported having family planning or acute diseases consultations but did not provide answers regarding the content of care.

¥¥¥¥ In women after C-section.

70.6% of women had medical consultations in postnatal period. During postnatal care for women, the majority had their blood pressure (92.9%) and temperature (87.2%) measured. Among those with cesarean sections, only 60.7% had their surgical scars examined during PNC visits. Additionally, 81.4% received advice on danger signs during the postnatal period, 74.2% on family planning, and 68.7% on breast care. However, fewer than half were advised on exercise (47.5%), reinitiating sexual activity (46.6%), and only 43.2% received advice on how to improve their emotional well-being. The median percentage of clinical activities performed for women was 66.7%, ranging from 0% to 100% (see Table 3).

### Perception of quality of postnatal care

**Fig 1:**
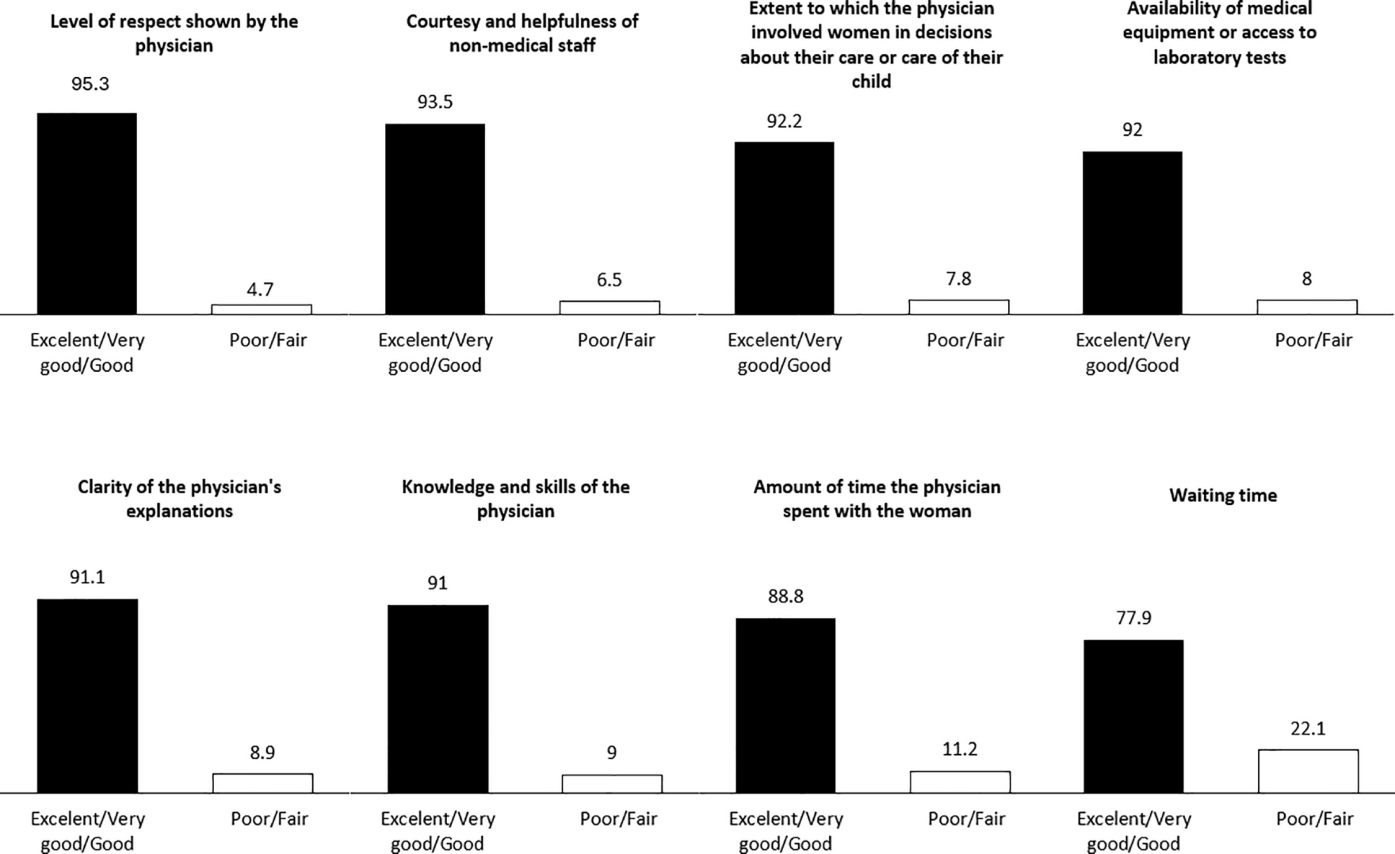
Perception of quality of postnatal care.

When asked to rate the quality of care received during the postnatal period, 95.3% of women rated the level of respect shown by their physician as excellent, very good, or good; 93.5% rated the courtesy and kindness of the staff at the family medicine clinic similarly. Furthermore, 92.2% of women felt that the physician effectively involved them in decisions regarding their care, while 92% rated the equipment and materials available for consultations as excellent, very good, or good. The clarity of the physician’s explanations and the physician’s knowledge and skills were rated as excellent, very good, or good by 91% of women. 88.8% rated the amount of time spent with the physician as excellent, very good, or good, while the waiting time was rated in the same way by only 77.9% (Fig 1). Overall, the median PNC perceived quality score was 25 points, with scores ranging between 10–40 points.

### Factors associated with perceived quality of postnatal care

Table 4 presents the factors associated with the perceived quality of PNC. Notably, being aged 35 years and older, receiving a higher number of clinical activities for their babies, and using private providers, were associated with higher perceived quality. In contrast, lower education levels, previous pregnancies, and poor self-reported mother and infant health were associated with lower perceived quality.

**Table 4:** Results of poisson multivariable regression for the factors associated with perceived quality of postnatal care, n = 885.

Variable	Adjusted prevalence ratio	Robust standard errors	95% CI	p
**General characteristics of mothers**				
Age ≥ 35 years	**1.06**	**0.03**	**1.002, 1.123**	**0.042**
**Schooling**				
Primary school or less	**0.93**	**0.02**	**0.89, 0.97**	**0.001**
Secondary school	**0.98**	**0.005**	**0.97, 0.99**	**<0.001**
**Marital status**				
Single/Divorced/Separated/Widowed	1.01	0.03	0.96, 1.07	0.665
Remunerated job	0.99	0.01	0.97, 1.02	0.727
With previous pregnancies	**0.98**	**0.002**	**0.97, 0.98**	**<0.001**
**Current health status**				
Mother’s perception of infant’s health as regular or poor	**0.91**	**0.03**	**0.85, 0.98**	**0.013**
One or more child’s danger signs	0.99	0.03	0.95, 1.05	0.921
Women’s self-rated health as regular or poor	**0.87**	**0.03**	**0.81, 0.93**	**<0.001**
Risk of postpartum depression	1.02	0.06	0.92, 1.14	0.677
**Healthcare during the postnatal period**				
Percentage of clinical activities provided for babies	**1.003**	**0.00006**	**1.0033, 1.0035**	**<0.001**
Percentage of clinical activities provided for women	**1.0005**	**0.0003**	**0.999, 1.001**	**0.055**
Private healthcare provider	**1.16**	**0.08**	**1.02, 1.32**	**0.026**
IMSS and other health care providers	1.002	0.04	0.92, 1.09	0.955

Dependent variable: perceived quality of care by women during postnatal period. Reference categories: < 35 years; high school or higher; unemployed; primipara; mother’s perception of infant’s health as excellent, very good or good, no infants warning signs; women’s self-rated health as excellent, very, good or good; without risk of postnatal depression; IMSS health care provider.

## Discussion

The present study found that one-third of women and 12% of infants in the IMSS maternal eCohort had no PNC visits in the 6 weeks following hospital discharge after childbirth. Most mothers and infants attended PNC at IMSS, with 17.7% using both IMSS and private care, and 10% using only private services. Infants received 82.4% of recommended care and mothers received 66.7% of recommended clinical actions during PNC visits. The lowest-rated aspects of care were wait times and time spent with providers. The median perceived quality score was 25 out of 40. Higher quality perceptions were associated with being over 35, receiving better infant care content, and receiving care from private providers, while lower education, previous pregnancies, and poor infant health were associated with lower quality perceptions.

PNC is essential for advancing progress toward the Sustainable Development Goals related to reproductive health, particularly in reducing maternal and neonatal mortality [1]. Yet, despite its importance, PNC remains the least prioritized intervention within the continuum of maternal and child healthcare [11]. This research found that almost three in ten women and one in ten infants lacked medical follow-ups during the postnatal period after hospital discharge. The highest number of visits for mothers and babies was three, consistent with WHO recommendations for at least three postnatal visits after discharge within 3 days, 7–14 days and 6 weeks following childbirth.

Using data from 48 low- and middle-income countries (4 in the Americas, 13 in Asia, and 31 in Africa), the Countdown to 2030 Collaboration found that 59% of mothers and 42% of infants received PNC [12]. Another study of 74 countries (1 in Europe, 2 in Oceania, 6 in the Americas, 18 in Asia, and 47 in Africa) found that 58% of mothers and 28% of infants received PNC [13]. In the present study, PNC coverage was 67% for mothers and 84% for infants. There are many reasons for higher rates of PNC use in our study including the fact that Mexico is a wealthier country that those included in the above-mentioned studies, the data is more recent, and the recruitment was performed in health facilities rather than in the general population. Nonetheless, poor PNC utilization is concerning, especially considering WHO recommendation for integrated postnatal care for mothers and infant dyads. In our study, the main reasons for not participating in postnatal consultations included the belief that neither the mother nor the infant required health care (40.3%), followed by difficulties in obtaining an appointment (19.4%), and lengthy waiting times (12.5%). These findings are similar to those reported by Bancalari in 2022 in Mexico [5], Zamawe in 2015 in Malawi [14], and Tesfahun in 2014 in Ethiopia [15], where many mothers reported a low perceived need for PNC when their newborns appeared healthy or when they believed the health issue would resolve without medical intervention. It is also in line with data from 20 countries (16 in Africa, 2 in Latin America, and 2 in Asia) [16], where the predominant reason for not attending PNC was attributed to long waiting times for appointments.

Technical quality, which includes disease- or condition-specific clinical actions, along with a respectful and supporting relationship between healthcare professionals and patients, are vital for high-quality care. WHO and PAHO emphasize the importance of not only life-saving interventions but also supporting the well-being of women and their infants through positive postnatal experiences and counseling [1]. In our study, the clinical activities required during the postnatal period were identified by the QuEST network expert group and IMSS clinical specialists, based on recommendations from WHO, PAHO, and official Mexican postnatal care standards [1,4]. Analysis of these activities identified that infants received 82.4% of recommended activities, while women received only 66.7%. The least performed clinical activities for infants were the physician’s recommendation on when to take the baby to the pediatrician, and how to interact with the baby, while many women were not advised about physical exercise, resuming sexual activity, or preventing postpartum depression. Currently, research primarily focuses on the first 48 hours after delivery, with limited studies investigating care during the subsequent six weeks, as outlined by the WHO. A relevant study from Ghana indicated that mothers often receive inadequate explanations about their postnatal care. In some instances, infants are separated from their mothers and taken to another room for clinical procedures, leaving mothers unaware of the activities being performed [17]. Therefore, our study provides crucial evidence regarding the clinical care that women and their babies receive in the 6 weeks following post-delivery discharge. We identified a significant need to promote the importance of postnatal consultations, counseling them on physical exercise, addressing the resumption of sexual activity, preventing and detecting postpartum depression, and providing guidance on childcare.

A positive postnatal experience is an essential component of high-quality care. The WHO characterizes a positive postnatal experience as one where women, newborns, and their families are provided with consistent information, reassurance, and support from healthcare staff, and where a well-resourced and adaptable health system acknowledges the needs of women and babies while honoring their cultural backgrounds [1]. In our research, the median perceived quality of PNC among women was 25 points on a scale of 8–40. Most women rated the respect from doctors and the courtesy of healthcare staff as excellent or good; however, satisfaction with waiting times and consultation lengths was lower. In earlier assessments of our cohort, we observed that the average waiting period to see a physician was 30 minutes [7]. Although waiting time is often overlooked in postnatal consultations, a qualitative study conducted in Zanzibar indicated that healthcare personnel believe patients avoid attending postnatal consultations due to extended waiting times, perceiving them as fatigued and feeling like their time is wasted [18]. Furthermore, long waiting times can be particularly frustrating for women attending with their newborns, due to the concern about exposing them to potential infections in the clinic, as highlighted in a study conducted in Ethiopia [19]. Regarding the duration of consultations it varies based on the specific needs of each woman and her newborn. Our study indicates that one in ten women rated the duration of their consultation as either regular or poor. Previous analysis of prenatal care of this cohort revealed an average consultation time of 20 minutes, with more than half of the women describing it as regular or poor [7]. Currently, there are no institutional or international guidelines recommending the duration of postnatal consultations. However, these consultations should be long enough for health professionals to complete all necessary clinical activities for the mother and her infant. They should also provide essential information, support, and an opportunity for mothers to address any questions they may have.

Our study found that a higher percentage of clinical activities provided to both the mother and baby during the postnatal period is linked to a better perception of healthcare quality. This finding is significant as it underscores the importance of necessary clinical activities not only for the health of the mother-child binomial, but also for enhancing perceived quality.

We found that those over 35 years old had a higher perception of quality. This might be attributed to the likelihood that IMSS health professionals are more attentive to older mothers due to their higher risk of postpartum complications [20,21], or it could be that older women tend to ask more questions and, as a result, obtain more information. This aligns with findings from Kenya, Nepal, and Uganda, indicating that older women are generally more skilled at seeking and advocating for high-quality postnatal care [22,3]. Additionally, using private health services was associated with a better perception of quality, corroborating studies from Mexico and Nepal, which showed that private healthcare users rated their quality of care higher than public sector users [23,24]. This is often attributed to shorter waiting times and more user-centered care in private facilities.

In our study, being multiparous was linked to a lower perception of quality, similar to findings from Sweden [25], where researchers found that primiparous women were more satisfied with the quality of postnatal care than multiparous women.

We also found that lower education was associated with a greater likelihood of perceiving lower quality of care. This is different from previous research indicating that individuals with lower education may have lower expectations and a poorer understanding of medical procedures, leading them to be less critical of their healthcare and be more satisfied compared to those with higher education [26]. Our finding suggests potential disparities in care among women with lower levels of education, highlighting the need to strengthen healthcare services for this population. One way to do this is by using clearer, simpler language that everyone can understand instead of complicated medical terminology and by explaining the clinical procedures provided.

Our research has several limitations. First, all data used in this study were self-reported by participants, that may be affected by recall bias. Also, women with more education or prior childbirth experience may better recall or understand PNC content and the counseling received. However, differential recall by less educated women might indicate that health providers must tailor their communication style and counseling intensity to the client’s level of education and comprehension. The survey collected data on postnatal care visits within the first 6 weeks postpartum. Recall bias may occur, with participants likely to remember recent visits more accurately than earlier ones, though the interval between consultations and interviews was no longer than one month. Furthermore, participants’ responses might be influenced by social desirability bias, providing answers they think the interviewer expects; however, the telephone interviews may encourage more honest responses by excluding nonverbal cues. Finally, the study focuses solely on postnatal care for IMSS affiliates and does not include details about Mexican women without IMSS.

## Conclusions

Strengthening postnatal care is essential to ensure that all women and newborns receive comprehensive services that are perceived as high quality. Key areas for improvement include promoting the value of postnatal care among both healthcare providers and women, reducing waiting times, and enhancing the content and duration of consultations.

## Abbreviations

IMSS: Mexican Institute of Social Security
PAHO: Pan American Health Organization
PNC: Postnatal care
QuEST: Quality Evidence for Health System Transformation network
WHO: World Health Organization
